# Unraveling atherosclerotic cardiovascular disease risk factors through conditional probability analysis with Bayesian networks: insights from the AZAR cohort study

**DOI:** 10.1038/s41598-024-55141-2

**Published:** 2024-02-22

**Authors:** Parya Esmaeili, Neda Roshanravan, Samad Ghaffari, Naimeh Mesri Alamdari, Mohammad Asghari-Jafarabadi

**Affiliations:** 1 Liver and Gastrointestinal Diseases Research Center, Tabriz University of Medical Sciences, Tabriz, Iran; 2 Department of Epidemiology and Biostatistics, Faculty of Health, Tabriz University of Medical Sciences, Tabriz, Iran; 3 Cardiovascular Research Center, Tabriz University of Medical Sciences, Tabriz, Iran; 4 Endocrine Research Center, Tabriz University of Medical Sciences, Tabriz, Iran; 5 Road Traffic Injury Research Center, Tabriz University of Medical Sciences, Tabriz, Iran; 6 Cabrini Research, Cabrini Health, Malvern, VIC 3144 Australia; 7 School of Public Health and Preventive Medicine, Faculty of Medicine, Nursing and Health Sciences, Monash University, Melbourne, VIC 3004 Australia; 8 Department of Psychiatry, School of Clinical Sciences, Faculty of Medicine, Nursing and Health Sciences, Monash University, Clayton, VIC 3168 Australia

**Keywords:** Cardiovascular diseases, Health care, Risk factors

## Abstract

This study aimed at modelling the underlying predictor of ASCVD through the Bayesian network (BN). Data for the AZAR Cohort Study, which evaluated 500 healthcare providers in Iran, was collected through examinations, and blood samples. Two BNs were used to explore a suitable causal model for analysing the underlying predictor of ASCVD; Bayesian search through an algorithmic approach and knowledge-based BNs. Results showed significant differences in ASCVD risk factors across background variables’ levels. The diagnostic indices showed better performance for the knowledge-based BN (Area under ROC curve (AUC) = 0.78, Accuracy = 76.6, Sensitivity = 62.5, Negative predictive value (NPV) = 96.0, Negative Likelihood Ratio (LR−) = 0.48) compared to Bayesian search (AUC = 0.76, Accuracy = 72.4, Sensitivity = 17.5, NPV = 93.2, LR− = 0.83). In addition, we decided on knowledge-based BN because of the interpretability of the relationships. Based on this BN, being male (conditional probability = 63.7), age over 45 (36.3), overweight (51.5), Mets (23.8), diabetes (8.3), smoking (10.6), hypertension (12.1), high T-C (28.5), high LDL-C (23.9), FBS (12.1), and TG (25.9) levels were associated with higher ASCVD risk. Low and normal HDL-C levels also had higher ASCVD risk (35.3 and 37.4), while high HDL-C levels had lower risk (27.3). In conclusion, BN demonstrated that ASCVD was significantly associated with certain risk factors including being older and overweight male, having a history of Mets, diabetes, hypertension, having high levels of T-C, LDL-C, FBS, and TG, but Low and normal HDL-C and being a smoker. The study may provide valuable insights for developing effective prevention strategies for ASCVD in Iran.

## Introduction

Atherosclerotic cardiovascular disease (ASCVD) is a chronic disorder that develops gradually during life, and its progress can be seen in the appearance of symptoms in patients^1^. Despite recent therapeutic advances, ASCVD remains the leading cause of death worldwide^2^. ASCVD is a pattern of atherosclerosis wherein the artery wall develops abnormalities called lesions. Depending on which artery is affected, ASCVD can lead to coronary heart disease, cerebrovascular disease and other peripheral vascular diseases, congestive heart failure, carotid heart disease, aneurysm, or kidney problems^3–5^. Ischemic heart disease and cerebrovascular disease are the first and third leading causes of death worldwide^5,6^.

ASCVD deaths are a main burden of death, accounting for more than 80% of all cases^7^. In China, ASCVD is the leading cause of death, with over 40% of deaths attributable to the disease, with increasing incidence^8^. Despite a decrease in the incidence of coronary heart disease in the United States over the past 30 years, ASCVD remains the leading cause of death among US residents, affecting approximately 5.2% of the population^9^. In Europe, ASCVD is responsible for approximately 3.9 million deaths annually, which accounts for 45% of all deaths^10^. In Iran, ASCVD is one of the main causes of death and disability, with the prevalence of risk factors for the disease increasing over the past few decades^11,12^. A global survey found that approximately 422.7 million people were living with ASCVD, with 17.9 million deaths due to the disease in 2015^13^. The global mortality rate for ASCVD is primarily due to population growth and aging and a lack of recognition and appropriate treatment of those with ASCVD risk factors^13,14^.

ASCVD is influenced by various risk factors, including dyslipidemia, inflammation, hypertension, diabetes, excessive consumption of sugar-sweetened beverages and alcohol, smoking, obesity, lifestyle, and associated conditions^15–17^. Of these, dyslipidemias, obesity, high glucose levels, high blood pressure, and insulin resistance are the most significant and common physiological and metabolic changes contributing to increased ASCVD mortality^18–22^. Long-term complications and mortality from ASCVD can be reduced by controlling modifiable risk factors such as maintaining a healthy diet, regular exercise, not smoking, and maintaining a healthy weight^23–25^. As such, taking a multifactorial approach to control ASCVD risk factors and modifying several overall risk factors, rather than just individual risk factors, is a more effective way to reduce ASCVD risks^26,27^.

Bayesian networks (BNs) constitute a pivotal facet of probabilistic models, embodying a robust probabilistic basis essential for managing uncertainty within artificial intelligence. Historically known as probabilistic causal networks^28^, BNs intricately utilize graph theory and probability theory to delineate associations among a given set of variables (depicted as nodes within the graph) and their conditional probabilities (CPs). While primarily recognized for their capacity to model and predict causal relationships, BNs extend their functionality to encapsulate and portray intricate probabilistic interdependencies among variables^28,29^. These interrelations manifest within directed acyclic graphs (DAGs), elucidating the directed dependencies and conditional associations amid variables^28,30,31^. Notably, BNs possess the capability to infer the probabilities of latent variables predicated upon known variables, thereby engendering a systemic alteration in the probabilities of all variables consequent to a change in a single variable's state. The continual evolution of BNs enables the systematic integration of data or domain-specific knowledge within the healthcare realm, and in particular for the case of our data, the risk factors of ASCVD, showcasing a burgeoning array of applications spanning diverse scientific domains, encompassing but not limited to medicine and the social sciences^32–35^.

On the other hand, the prevention of ASCVD mortality is a global public health priority^2,27^, and this necessitates the application of BNs for cardiovascular disease (CVD) risk prediction, diagnosis, evaluation, and clinical decision-making^36–38^. In this study, we aim to explore the most dominant risk factors of ASCVD that affect a population of healthcare providers at Tabriz University of Medical Sciences through BNs.

## Methods

### Study population

The data were collected as a part of the larger AZAR Cohort Study, prospective epidemiological research conducted by the liver and gastrointestinal diseases research center of Tabriz University of Medical Sciences (TBZMED) in Iran^39^. The cohort aims to evaluate 3000 participants, including healthcare providers in hospitals, schools, and health networks of TBZMED. In 2020, a total of 500 persons participated in this study in a cross-sectional manner^40^.

### Data collection

Our study involved conducting face-to-face health interviews and health examinations of full-time and long-term healthcare providers aged 18–75 years in hospitals, schools, and health networks of TBZMED. We excluded individuals who were pregnant or breastfeeders, or planned to retire within the next five years, and those with a history of debilitating psychiatric disorders or physical illnesses reported by a health professional. Participants provided information on their demographic characteristics, such as age, sex, marital status, and education level, as well as behavioral factors, including smoking status, and self-reported family history. All eligible healthcare providers, official staff, and lecturers of TBZMED were invited to participate in the study.

### Ethical approval

All participants were informed of the study purpose before they gave consent to participate, and then filled and signed the informed consent and assent. The Institutional Review Board (IRB) of TBZMED approved the study (ethics code: IR.TBZMED.REC.1400.1006).

### Measurements

Anthropometric measurements were performed according to the international standards for anthropometric assessment^41^ by trained technicians. Body weight was measured to the nearest 0.1 kg using an electronic scale (Seca 700 scale, Seca gmbh, Hamburg), while height was measured to the nearest 0.5 cm using a stadiometer (Seca 220 Telescopic Height Rod for Column Scales, Seca gmbh, Hamburg). Waist circumference (WC) was measured at the halfway point between the lower costal border and the iliac crest using a flexible steel tape (Lufkin Executive Thinline W 606, precision 1 mm)^42^. Body mass index (BMI) was calculated as weight (kg) divided by height (m) squared (kg/m^2^), and BMI values were categorized according to the World Health Organization's (WHO) criteria^43^. Serum samples were collected to assess total cholesterol (T-C), high-density lipoprotein cholesterol (HDL-C), fasting blood sugar (FBS), and triglycerides (TG). These samples were analyzed using Miura One automated equipment (I.S.E., Rome, Italy) and a commercial DiaSys kit (DiaSys Diagnostic Systems, Hamburg, Germany)^44^. Low-density lipoprotein cholesterol (LDL-C) was calculated using the Fried Ewald equation^45^. Metabolic syndrome (Mets) is a cluster of risk factors, including central obesity, high blood pressure, high blood sugar, and abnormal cholesterol levels. The diagnosis of Mets is based on the presence of three or more of these risk factors. The metabolic health status of each participant was defined based on the adult treatment panel-3 definition of Mets^46^.

### Study variables

The participants were classified based on various risk factors level, including (non-ASCVD vs with ASCVD)^47^, (sex male vs female), (age under45 vs with over45)^48^, (non-smokers vs Past or current smokers), (without diabetes vs with diabetes), (without hypertension vs with hypertension), and (without Mets vs with Mets). Participants were classified depending on their BMI: normal weight (BMI ranges from 18.5 to 25 kg/m^2^), overweight (BMI ranges from 25.01 to 30 kg/m^2^), and obesity (BMI ranges from ≥ 30.01 kg/m^2^)^49^. LDL-C (mg/dL), FBS (mg/dL), T-C (mg/dL), and TG (mg/dL) levels were categorized as follows: LDL-C level^49^: normal (< 130 mg/dL), and high (≥ 160 mg/dL), FBS level^51^: normal (≤ 99 mg/dL), and high (≥ 100 mg/dL), TG level^51^: normal (< 150 mg/dL), and high (≥ 150 mg/dL), and T-C level^49^: normal (< 200 mg/dL), and high (≥ 200 mg/dL). Also, HDL-C (mg/dL) was separated into the following three levels: HDL-C level: low (< 45 mg/dL), normal (45–55 mg/dL), and high (> 55 mg/dL)^50^.

### Bayesian networks

The BN models were developed and evaluated using a two-stage process, including (1) structural learning to determine the topology of the BN or DAG, and (2) parametric learning or estimation of CPs among the nodes, once the network topology was established. In our study, BN provided insight into how a group of ASCVD risk factors can influence the probability of occurring ASCVD, independent of sample size^53^. Our BNs are graphs with arcs linking nodes and no directed cycles, where our ASCVD risk factors and outcome variables are represented as nodes, and conditional dependencies between them are represented by directed edges or arrows^52^. Each node is associated with a CP table, which specifies the CP of each of its values for each combination of the parents' values^53,54^. Our procedure in BN modelling was to learn a BN structure by amalgamating algorithmic potency and expert insights with empirical evidence obtained through a systematic literature review. This rigorous approach ensures the relevance and significance of chosen variables in capturing intricate relationships and dependencies within the modeled system. This aligns with established research, as exemplified in studies by Ordovas et al.^32^ and Huang et al.^55^, which advocate for incorporating prior expert knowledge and comprehensive literature reviews in BN variable selection processes^32,55^. In other words, we decided to train two BNs; Bayesian search through an algorithmic approach and knowledge-based BN.

### Structures from the literature

We aimed to predict the most suitable causal model for analyzing variables related to ASCVD, which models underlying risk factors of ASCVD, including age, sex, diabetes, smoking status, hypertension, BMI, FBS, T-C, LDL-C, HDL-C, and TG. To achieve this, we utilize BN and select the probabilistic models for our purposes by an amalgamation of algorithmic search and knowledge-based models^32^.

**Figure 1:**
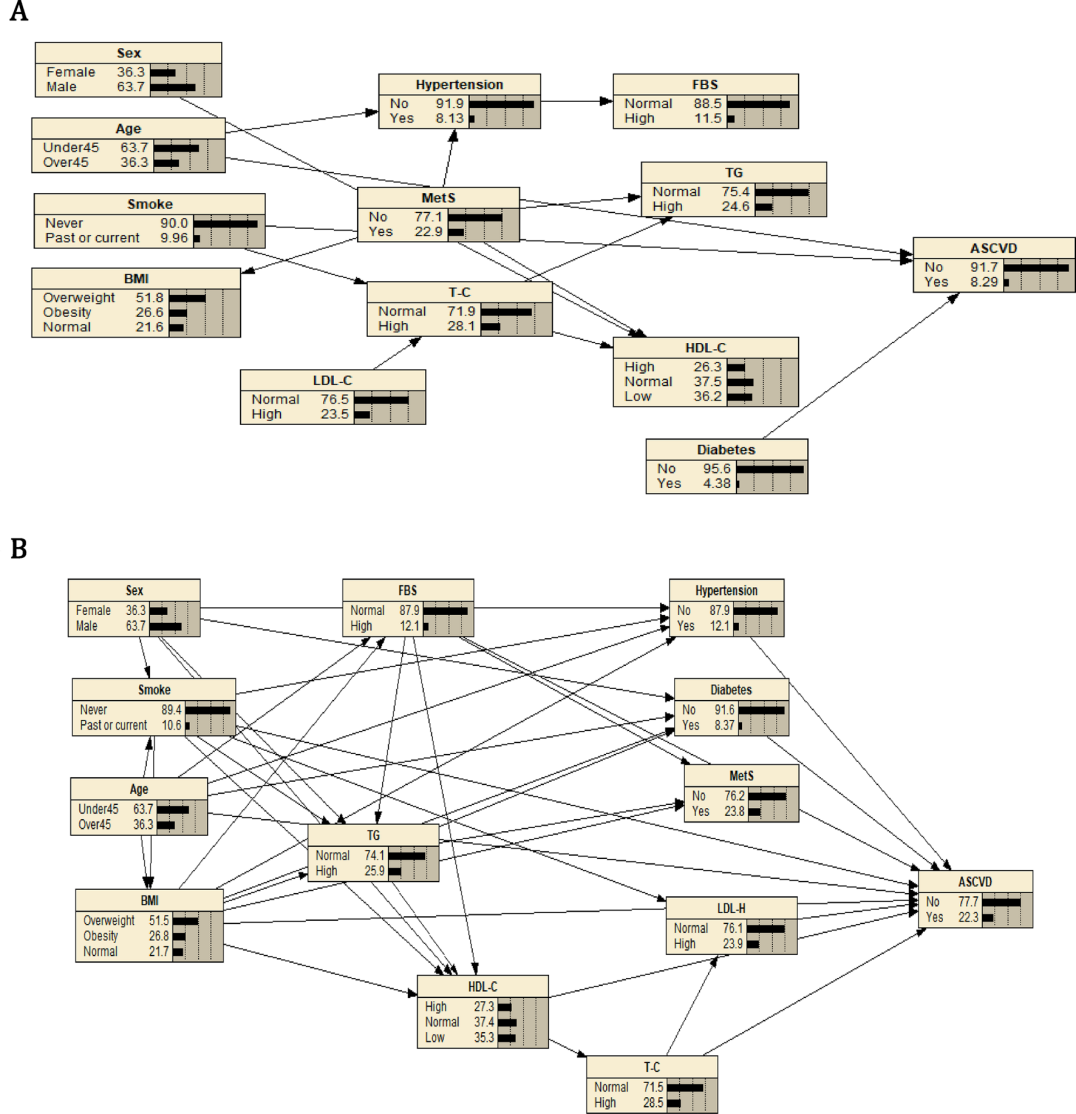
BN structures and probabilities for ASCVD.

The BN structure in Fig. 1 illustrates the interconnections between these variables and their impact on ASCVD risk. The obtained structure of the algorithmic search network, as depicted in Fig. 1A, reveals the factors that influence ASCVD. Notably, age, smoking, and diabetes have a significant impact on ASCVD probability, Fig. 1A includes direct links between these predictors and ASCVD. Additionally, Age → Hypertension → FBS, since hypertension is non-conditional, then FBS and age would be d-connected.

The knowledge-based network structure depicted in Fig. 1B provides a concise overview of the factors influencing ASCVD via a systematic search in the literature. The network highlights the connections between modifiable and non-modifiable risk factors in predicting various ASCVD conditions. Notably, TC, TG, HDL-C, and FBS are indirectly associated with ASCVD through other risks^14,29,56^. However, sex is indirectly associated with ASCVD through diabetes (Sex → Diabetes → ASCVD). Also, the path between TG and ASCVD is blocked after conditioning on HDL-C, a d-separation. TG is conditionally independent of ASCVD given HDL-C (TG → HDL-C → ASCVD), another example of a d-separation in this model. On the other hand, ASCVD is directly associated with smoking, hypertension, diabetes, BMI, HDL-C, LDL-C, cholesterol, and FBS^57,58^. Ultimately, our goal is to provide a precise and easily understandable prediction of ASCVD risk by analyzing the relationships between variables^29,62^.

By models we assumed d-separation, and the model consists of a set of independent predictors that lead to the outcome variable. D-separation is a criterion used in BN to determine whether two sets of variables are independent of each other given a third set of variables, and conditional independence between variables can be directly inferred from the graph using the d-separation criterion^59–61^.

### Statistical analysis

The Bayes search BN was built using GeNIe Academic Version 4.1.3402.0 (Built on 2023-10-03; License ID: 6c8hwje30dfnjbukdej30zg76), and the knowledge-based BN was built utilizing Netica 6.05 (Norsys software corp, USA)^63^, and the BNs were drawn using Netica. Categorical data were presented as count (percentage), and P-values were computed using Fisher’s exact test. We compared our 2 different structures using Akaike Information Criteria (AIC), and Bayesian Information Criteria (BIC) values. A smaller AIC and BIC value indicate a better structure. Furthermore, diagnostic indices including sensitivity, accuracy, specificity, positive predictive value (PPV), negative predictive value (NPV), positive likelihood ratio (LR+), negative likelihood ratio (LR−), and particularly area under the ROC curve (AUC) were calculated for comparing the BNs. We used the leave-one-out cross-validation procedure, as a standard method, to compute the AUC, accuracy, and diagnostic indices. Finally, based on the best-suited BN structure, CPs ASCVD and non-ASCVD were calculated in the datasets.

### Consent to participate

All participants filled out and signed the informed consent and assent. The participants' privacy was preserved. All methods were carried out according to relevant guidelines and regulations.

## Results

Out of 500 participants, 491 (98.2%) completed the study with a mean age of 43.2 (SD: 7.2, min–Max: 24.0–67.0) years and a prevalence of ASCVD equals to 7.7% (95%CI: 5.5–10.5). ASCVD was exclusively observed in males (100%) and patients aged over 45 years (89.4%). The majority of participants did not have dyslipidemia, with 50.0%, 52.6%, and 55.3% showing normal LDL-C, T-C, and TG. Most risk factors were within normal range with 78.9%, 63.2%, 42.1%, 84.6%, and 81.6% of participants having normal FBS, hypertension, and diabetes. Fisher’s exact tests revealed significant differences (P < 0.05) between the ASCVD and non-ASCVD groups for sex, age, diabetes, smoke, Mets, TG, T-C, LDL-C, and HDL-C, but not for hypertension, BMI, and FBS (all p < 0.05). Also, older individuals had 21.7% (95%CI: 15.3–29.1) higher ASCVD rates compared to younger ones. Similarly, males exhibited 12.1% (8.3–16.2) higher rates compared to females. Individuals with past or current smoking demonstrated 14.4% (4.9–28.0) higher ASCVD rates compared to individuals who have never smoked (all p < 0.05). Individuals with diabetes had 28.4% (11.3–50.2) higher ASCVD rates compared to those without diabetes. In individuals with Mets, the ASCVD rate was found to be 10.5% (4.3–18.5). Additionally, individuals with normal levels of TG, T-C, HDL-C, and LDL-C had 8.5%, 7.3%, 9%, and 11.3% (2.6–16.1, 1.8–14.1, 0.4–4.1, and CI: 5.1–19.3) higher ASCVD rates, respectively, compared to those with high levels of TG, T-C, HDL-C, and LDL-C (All p < 0.05). Refer to Table 1 for more detailed participant information.

**Table 1:** Sociodemographic and clinical characteristics in the study population ASCVD cases and non-ASCVD.

Variables	States	All* (n = 491)	ASCVD (n = 38)	Non-ASCVD (n = 453)	Percent Difference (CI)	p-value ‡
Age (years)						< 0.001
	Under45	319 (63.8)	4 (10.5)	309 (68.1)	Reference	
	Over45	181 (36.2)	34 (89.5)	114 (31.9)	21.7 (15.3 to 29.1)	
Sex						< 0.001
	Female	181 (36.2)	0 (0.0)	178 (39.3)	Reference	
	Male	319 (63.8)	38 (100)	275 (60.7)	12.1 (8.3 to 16.2)	
Diabetes						< 0.001
	No	479 (95.8)	31 (81.6)	440 (97.1)	Reference	
	Yes	21 (4.2)	7 (18.4)	13 (2.9)	28.4 (11.3 to 50.2)	
Smoking						< 0.001
	Never	445 (89.0)	28 (73.7)	409 (91.5)	Reference	
	Past or current	49 (9.8)	10 (26.3)	38 (8.5)	14.4 (4.9 to 28.0)	
Hypertension						0.053
	No	462 (92.4)	32 (84.6)	421 (92.9)	Reference	
	Yes	38 (7.6)	6 (15.4)	32 (7.1)	8.7 (− 0.1 to 23.5)	
Mets n (%)						< 0.001
	No	386 (77.2)	20 (52.6)	357 (78.8)	Reference	
	Yes	114 (22.8)	18 (47.4)	96 (21.2)	10.5 (4.3 to 18.5)	
BMI (kg/m^2^)						0.548
	Normal	107 (21.4)	7 (17.9)	99 (21.7)	Reference	
	Overweight	257 (51.4)	18 (46.2)	235 (52.5)	0.5 (− 6.4 to 5.6)	
	Obesity	133 (26.6)	13 (35.9)	118 (25.8)	3.3 (− 4.2 to 10.5)	
FBS (mg/dL)						0.051
	Normal	444 (88.8)	30 (78.9)	405 (89.6)	Reference	
	High	56 (11.2)	8 (21.1)	48 (10.4)	7.4 (0.0 to 19.0)	
TG (mg/dL)						0.002
	Normal	379 (75.8)	21 (55.3)	350 (77.3)	Reference	
	High	121 (24.2)	17 (44.7)	103 (22.7)	8.5 (2.6 to 16.1)	
T-C (mg/dL)						0.007
	Normal	360 (72.0)	20 (52.6)	332 (73.3)	Reference	
	High	140 (28.0)	18 (47.4)	121 (26.7)	7.3 (1.8 to 14.1)	
HDL-C (mg/dL)						0.013
	Normal	187 (37.4)	10 (26.3)	172 (38.4)	Reference	
	Low	180 (36.0)	22 (57.9)	155 (34.1)	6.9 (0.1 to 13.1)	
	High	133 (26.6)	6 (15.8)	126 (27.5)	0.9 (0.4 to 4.1)	
LDL-C (mg/dL)						< 0.000
	Normal	383 (76.6)	19 (50.0)	356 (78.6)	Reference	
	High	117 (23.4)	19 (50.0)	97 (21.4)	11.3 (5.1 to 19.3)	

ASCVD, atherosclerotic cardiovascular disease; Mets, metabolic syndrome; BMI, body mass index; FBS, fasting blood pressure; TG, triglyceride; T-C, total cholesterol; HDL, high-density lipoprotein; LDL, low-density lipoprotein; CI, confidence interval.

*Numbers are expressed as frequency (percent).

^‡^χ^2^ test for the difference between ASCVD and non-ASCVD cases.

We evaluated the quality of the BN models using AIC and BIC measures, and the results are summarized in Table 2. Lower values of AIC and BIC are indicative of a better model fit. The results suggest that knowledge-based BN with lower AIC and BIC could be considered an appropriate representation of the data.

**Table 2:** AIC and BIC values for comparing the different BN structures.

Models	AIC	BIC
Bayes search BN	6870.8	6921.4
Knowledge-based BN	6729.7	6780.3

AIC, akaike information criteria; BIC, Bayesian information criteria; BN, Bayesian network.

**Figure 2:**
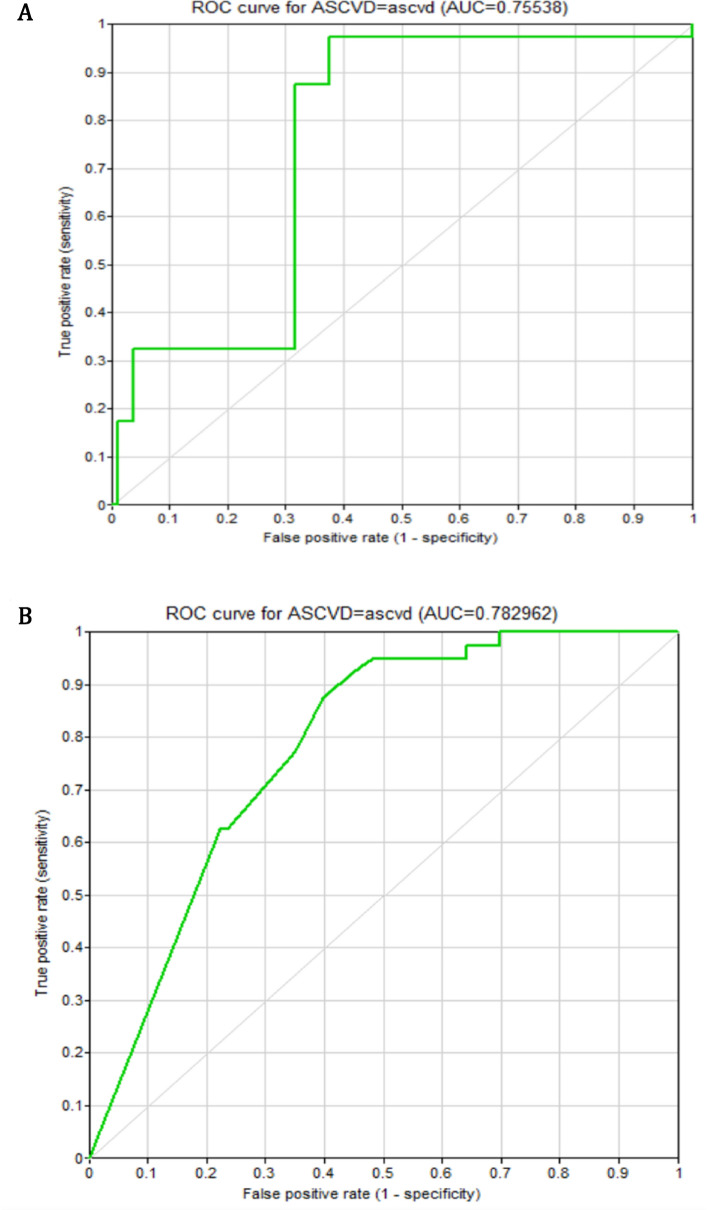
Receiver operating characteristics curves of the Bayesian Networks.

Figure 2 shows ROC curves of various BN models under different methods: (1) BN constructed by Algorithmic search network structure; (2) BN constructed by Knowledge-based network structure.

### Predictive performance of BN models

**Figure 3:**
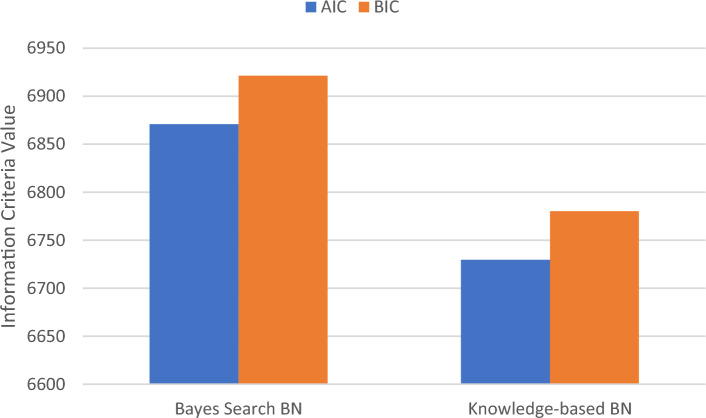
Model comparisons.

The diagnostic indices showed better performance for the knowledge-based BN (AUC = 0.78, Accuracy = 76.6, Sensitivity = 62.5, NPV = 96.0, LR− = 0.48) compared to Bayesian search (AUC = 0.76, Accuracy = 72.4, Sensitivity = 17.5, NPV = 93.2, LR− = 0.83). However, knowledge-based BN performed subordinately compared to Bayesian search in terms of specificity (77.8 vs 98.9), PPV (19.7 vs 58.3), and LR + (2.8 vs 16.1) (Fig. 3).

Generally, we decided on knowledge-based BN as the better-performing model regarding its better diagnostic indices and lower values of AIC and BIC compared to the Bayesian search. Additionally, as the arrows for knowledge-based BN were obtained based on a systematic search from the literature, clinical interpretability of the relationships in knowledge-based BN is guaranteed (Table 3).

**Table 3:** Diagnostic indices of BN models.

Models	AUC	Accuracy	Sensitivity	specificity	NPV	PPV	LR+	LR−
Bayes search BN	75.5	72.4	17.5	98.9	93.2	58.3	16.1	83.4
Knowledge-based BN	78.2	76.6	62.5	77.8	96.0	19.7	2.8	48.2

PPV, positive predictive value; NPV, negative predictive value; LR +, positive likelihood ratio; LR−, negative likelihood ratio; AUC, area under ROC curve; BN, Bayesian network.

### Conditional probabilities of the Bayesian network

CPs of ASCVD based on knowledge-based BN structure are shown in Table 4. The CP of developing ASCVD using smoke given hypertension is equal to 18.4%. The findings revealed a varying CP of ASCVD occurrence associated with different BMI levels, conditioned on diabetes equal to 10.3%. Also, the CP of ASCVD occurrence about age, conditional on hypertension equal to 11.7%. Notably, the CP of ASCVD for FBS given hypertension is equal to 13.1%. Also, CPs of other variables based on knowledge-based BN structure are shown in Table S1.

**Table 4:** Conditional probabilities of BN for ASCVD & non-ASCVD once variables are instantiated to different values.

Variables		Conditional probability for non-ASCVD	Conditional probability for ASCVD
Smoke	Hypertension	87.0	13.0
Smoke	BMI	89.4	10.6
Smoke	LDL-C	81.6	18.4
Smoke	HDL-C	94.3	5.7
BMI	HDL-C	95.4	4.6
BMI	Diabetes	89.7	10.3
Age	Diabetes	89.8	10.2
Age	Hypertension	88.3	11.7
Age	FBS	96.6	3.6
HDL-C	T-C	94.6	6.0
FBS	Hypertension	86.9	13.1
FBS	HDL-C	93.1	6.9

The CPs are obtained at the presence or higher risk levels of the particular variables; for example: Conditional probability for non-ASCVD = 87% is obtained at smoking = yes and Hypertension = Yes.

BN, Bayesian Network; ASCVD, atherosclerotic cardiovascular disease; Mets, metabolic syndrome; BMI, body mass index; FBS, fasting blood pressure; TG, triglyceride; T-C, total cholesterol; HDL, high-density lipoprotein; LDL, low-density lipoprotein.

### Strength of influence of the relationship

The strength of the relationship between variables in Table 5 indicates that in knowledge-based BN, variables such as diabetes (0.017), hypertension (0.016), FBS (0.016), and LDL-C (0.016) have the greatest influence on the ASCVD variable. For more details on the strength of the relationships among BNs, refer to Table S2.

**Table 5:** Strength of influence of the relationship in knowledge-based BN.

From	To	Average
Age	FBS	0.171
Age	ASCVD	0.011
Age	Diabetes	0.088
Age	BMI	0.076
Age	Hypertension	0.103
Age	Smoke	0.013
BMI	FBS	0.147
BMI	Mets	0.224
BMI	ASCVD	0.013
BMI	Diabetes	0.126
BMI	TG	0.166
BMI	Hypertension	0.102
BMI	HDL-C	0.142
T-C	LDL-C	0.624
T-C	ASCVD	0.016
Diabetes	ASCVD	0.017
FBS	TG	0.253
FBS	Hypertension	0.167
FBS	Mets	0.271
FBS	HDL	0.148
FBS	ASCVD	0.016
HDL-C	T-C	0.199
HDL-C	ASCVD	0.012
Hypertension	ASCVD	0.017
LDL-C	ASCVD	0.017
Sex	HDL-C	0.168
Sex	Smoke	0.148
Sex	Diabetes	0.104
Sex	Hypertension	0.122
Sex	TG	0.198
Smoke	LDL-C	0.116
Smoke	ASCVD	0.016
Smoke	BMI	0.121
Smoke	Hypertension	0.194
Smoke	TG	0.251
Smoke	HDL-C	0.161
TG	Diabetes	0.162
TG	Mets	0.272
TG	HDL-C	0.143

ASCVD, atherosclerotic cardiovascular disease; Mets, metabolic syndrome; BMI, body mass index; FBS, fasting blood pressure; TG, triglyceride; T-C, total cholesterol; HDL, high-density lipoprotein; LDL, low-density lipoprotein.

## Discussion

In our study of 491 participants, ASCVD was exclusively observed in males and patients aged over 45 years, with a prevalence of 7.7%. Our BN models showed a good fit, and their predictive performance for ASCVD risk factors was accurate. The CPs revealed that being male, aged over 45, having diabetes, Mets, and other risk factors increased ASCVD risk, while high HDL-C reduced it. These results provide valuable insights into ASCVD risk factors and can aid in developing effective prevention, predicting various conditions, supporting health research, and determining relevant findings.

BN models have been increasingly used in the field of cardiovascular risk prediction due to their ability to model complex relationships among risk factors and incorporate prior knowledge into the model. Several studies have demonstrated the effectiveness of BN models in predicting cardiovascular risk, such as predicting coronary heart disease risk in Korean adults^64^, identifying important risk factors for stroke in the Chinese population^65^, and predicting major cardiovascular events in patients with hypertension^66^. These studies highlight the potential of BN models in improving CVD risk prediction and helping clinicians make more informed decisions.

In one study, two BN models were developed to predict ASCVD risk using data from a large population-based cohort. The model included demographic factors, ASCVD risk factors, and their inter-relationships. The performance of the models was evaluated using various measures, including sensitivity, specificity, accuracy, PPV, NPV, LR+, LR−, and AUC. The results showed that the knowledge-based BN model had good predictive performance, and identified several risk factors associated with ASCVD, such as age, sex, smoking status, hypertension, and lipid levels. Overall, the BN approach provides a promising tool for predicting cardiovascular risk and can aid in the development of personalized prevention strategies^67^.

The finding that ASCVD was exclusively observed in males and patients aged over 45 years is consistent with previous research on cardiovascular risk factors. Multiple studies have identified being male and higher age as independent risk factors for ASCVD^68–70^. This may be due to hormonal differences between men and women, as well as changes in the cardiovascular system that occur with aging, such as endothelial dysfunction and arterial stiffening^71,72^. It is important to note, however, that the present study did not identify age or sex as significant predictors of ASCVD in the BN models. This may be due to the complex interplay between multiple risk factors and the non-linear relationships between them. Further research is needed to fully understand the relative contributions of age, sex, and other risk factors to the development of ASCVD.

The results of the study suggest that several traditional risk factors, including diabetes and Mets, are associated with an increased risk of ASCVD. This finding is consistent with previous research that has identified diabetes as a strong predictor of CVD^73^. Mets, which is characterized by a cluster of metabolic abnormalities including abdominal obesity, dyslipidemia, and insulin resistance, has also been shown to be a strong predictor of CVD^74^. In addition to these risk factors, the study found that high HDL-C was associated with a reduced risk of ASCVD. This is consistent with previous research that has identified HDL-C as a protective factor against CVD^75^. The findings of this study underscore the importance of identifying and managing traditional risk factors for ASCVD, as well as the potential benefit of interventions to increase HDL-C levels.

### Strengths and limitations

Strengths of this study include employing the BN models, which allow for the modelling of complex relationships among various risk factors and the incorporation of prior knowledge into the model. Additionally, the study identified several traditional risk factors associated with an increased risk of ASCVD, such as diabetes and Mets, as well as a protective factor, high HDL-C. The study provides valuable insights into ASCVD risk factors and can aid in developing effective prevention and management strategies, and facilitate treatment.

One potential limitation of the study is the small sample size of 491 participants, which may limit the generalizability of the findings to larger populations. Future studies with larger sample sizes could help confirm the results and identify additional risk factors associated with ASCVD. The absence of ASCVD among female participants in this study can be attributed to several factors, including the low number of women in this study limiting the generalizability of the findings. Additionally, this study exclusively focused on healthcare providers, which may have influenced the lack of ASCVD cases among female participants. Another limitation is the lack of inclusion of certain risk factors, such as family history, which may be important predictors of ASCVD. Future studies could incorporate additional risk factors into the model to improve its accuracy. Another shortcoming of the study is having to discretise continuous variables into categorical variables for the BN models. Though clinical guidelines are much better written with categorical variables, however, this brings some loss of information in the model when discretising continuous variables. Future direction is recommended to assess how this process affects the statistical information provided in the BN models. As well as finding ways to incorporate the continuous variables into BN models.

## Conclusion

In conclusion, the study provides valuable insights into ASCVD risk factors and demonstrates the potential of BN models in predicting cardiovascular risk in a large population-based cohort. The BN models showed good fit and accurate predictive performance for ASCVD risk factors including age, sex, smoking status, hypertension, lipid levels, diabetes, and Mets. The study found that high HDL-C was associated with a reduced risk of ASCVD. The findings underscore the importance of identifying, preventing, and managing traditional risk factors for ASCVD, as well as the potential benefit of interventions to increase HDL-C levels. Overall, the BN approach provides a promising tool for predicting cardiovascular risk and can aid in the development of personalized prevention strategies and health policymaking. However, the study's limitations, including a small sample size, and the complexity of the interplay between risk factors should be taken into account when interpreting the results. Further research is needed to fully understand the complex interplay between multiple risk factors and non-linear relationships between them, as well as to validate the study findings and improve our understanding of ASCVD risk factors.

### Supplementary Information

Supplementary Table S1.

Supplementary Table S2.

## Data Availability

The datasets generated during and/or analysed during the current study are available from the corresponding author upon reasonable request.
