# Supplementary material for: Unraveling atherosclerotic cardiovascular disease risk factors through conditional probability analysis with Bayesian networks: insights from the AZAR cohort study
Source: Sci Rep. 2024 Feb 22;14:4361. doi: 10.1038/s41598-024-55141-2 (PMC10883955; doi:10.1038/s41598-024-55141-2)
Supplement: Supplementary file 1 — Supplementary Table S1. [file 41598_2024_55141_MOESM1_ESM.docx]

**Table S1. Conditional probabilities for BN for ASCVD & non-ASCVD once variables are instantiated to different values**

| **Variables** | | **Conditional probability for non-ASCVD** | **Conditional probability for ASCVD** |
| --- | --- | --- | --- |
| **Smoke** | **Hypertension** |  |  |
| Never | Yes | 50 | 50 |
|  | No | 96.6 | 3.4 |
| Past or current | Yes | 50 | 50 |
|  | No | 50 | 50 |
| **Smoke** | **BMI** |  |  |
| Never | Normal | 92.9 | 7.1 |
|  | Overweight | 96.5 | 3.5 |
|  | Obesity | 87.5 | 12.5 |
| Past or current | Normal | 66.7 | 33.3 |
|  | Overweight | 50 | 50 |
|  | Obesity | 50 | 50 |
| **Smoke** | **LDL-C** |  |  |
| Never | Normal | 96.6 | 3.4 |
|  | High | 50 | 50 |
| Past or current | Normal | 50 | 50 |
|  | High | 50 | 50 |
| **Smoke** | **HDL-C** |  |  |
| Never | Low | 97.6 | 2.4 |
|  | Normal | 97.6 | 2.4 |
|  | High | 96.6 | 3.4 |
| Past or current | Low | 97.6 | 3.4 |
|  | Normal | 97.6 | 2.4 |
|  | High | 96.6 | 3.4 |
| **BMI** | **HDL-C** |  |  |
| Normal | Low | 93.8 | 6.2 |
|  | Normal | 95.7 | 4.3 |
|  | High | 92.9 | 7.1 |
| Overweight | Low | 97.4 | 2.6 |
|  | Normal | 97.6 | 2.4 |
|  | High | 96.6 | 3.4 |
| Obesity | Low | 96.4 | 3.6 |
|  | Normal | 92.3 | 7.7 |
|  | High | 87.5 | 12.5 |
| **BMI** | **Diabetes** |  |  |
| Normal | Yes | 92.9 | 7.1 |
|  | No | 92.9 | 7.1 |
| Overweight | Yes | 96.6 | 3.4 |
|  | No | 96.6 | 3.4 |
| Obesity | Yes | 87.5 | 12.5 |
|  | No | 87.5 | 12.5 |
| **Age** | **Diabetes** |  |  |
| Under45 | Yes | 50 | 50 |
|  | No | 96.5 | 3.4 |
| Over45 | Yes | 50 | 50 |
|  | No | 87.5 | 12.5 |
| **Age** | **Hypertension** |  |  |
| Under45 | Yes | 50 | 50 |
|  | No | 96.5 | 3.5 |
| Over45 | Yes | 50 | 50 |
|  | No | 96.5 | 3.5 |
| **Age** | **FBS** |  |  |
| Under45 | Normal | 96.5 | 3.5 |
|  | High | 50 | 50 |
| Over45 | Normal | 87.5 | 12.5 |
|  | High | 66.7 | 33.3 |
| **HDL-C** | **T-C** |  |  |
| Low | Normal | 97.6 | 2.4 |
|  | High | 75 | 25 |
| Normal | Normal | 97.6 | 2.4 |
|  | High | 75 | 25 |
| High | Normal | 96.6 | 3.4 |
|  | High | 85.7 | 14.3 |
| **FBS** | **Hypertension** |  |  |
| Normal | Yes | 50 | 50 |
|  | No | 96.6 | 3.4 |
| High | Yes | 50 | 50 |
|  | No | 50 | 50 |
| **FBS** | **HDL-C** |  |  |
| Normal | Low | 97.4 | 2.6 |
|  | Normal | 97.6 | 2.4 |
|  | High | 96.6 | 3.4 |
| High | Low | 66.7 | 33.3 |
|  | Normal | 75 | 25 |
|  | High | 50 | 50 |

BN: Bayesian Network; ASCVD: Atherosclerotic Cardiovascular Disease; MetS: Metabolic Syndrome; BMI; Body Mass Index; FBS: Fasting Blood Pressure; TG: Triglyceride; T-C: Total Cholesterol; HDL: High-Density Lipoprotein; LDL: Low-Density Lipoprotein.

The CPs are obtained at the presence or higher risk levels of the particular variables; for example: Conditional probability for non-ASCVD=87% is obtained at smoking=yes and Hypertension=Yes.
