# Supplementary material for: Unraveling atherosclerotic cardiovascular disease risk factors through conditional probability analysis with Bayesian networks: insights from the AZAR cohort study
Source: Sci Rep. 2024 Feb 22;14:4361. doi: 10.1038/s41598-024-55141-2 (PMC10883955; doi:10.1038/s41598-024-55141-2)
Supplement: Supplementary file 2 — Supplementary Table S2. [file 41598_2024_55141_MOESM2_ESM.docx]

**Table S2. Strength of influence of the relationship in Bayesian search model**

| **From** | **To** | **Average** |
| --- | --- | --- |
| Age | Hypertension | 0.099 |
| Age | ASCVD | 0.273 |
| T-C | HDL-C | 0.351 |
| T-C | TG | 0.253 |
| Diabetes | ASCVD | 0.308 |
| Hypertension | FBS | 0.136 |
| LDL-C | T-C | 0.658 |
| MetS | BMI | 0.312 |
| MetS | Hypertension | 0.131 |
| MetS | HDL-C | 0.333 |
| MetS | TG | 0.402 |
| Sex | HDL-C | 0.342 |
| Smoke | T-C | 0.238 |
| Smoke | ASCVD | 0.242 |

ASCVD: Atherosclerotic Cardiovascular Disease; MetS: Metabolic Syndrome; BMI; Body Mass Index; FBS: Fasting Blood Pressure; TG: Triglyceride; T-C: Total Cholesterol; HDL: High-Density Lipoprotein; LDL: Low-Density Lipoprotein.
